# All-Optical Rotational Ultrasound Imaging

**DOI:** 10.1038/s41598-019-41970-z

**Published:** 2019-04-03

**Authors:** Richard J. Colchester, Callum Little, George Dwyer, Sacha Noimark, Erwin J. Alles, Edward Z. Zhang, Christopher D. Loder, Ivan P. Parkin, Ioannis Papakonstantinou, Paul C. Beard, Malcolm C. Finlay, Roby D. Rakhit, Adrien E. Desjardins

**Affiliations:** 1 Department of Medical Physics and Biomedical Engineering, University College London, Malet Place Engineering Building, London, WC1E 6BT UK; 2 Wellcome/EPSRC Centre for Interventional and Surgical Sciences, University College London, Charles Bell House, 67-73 Riding House Street, London, W1W 7EJ UK; 3 Department of Cardiology, Royal Free Hampstead NHS Trust, Pond Street, London, NW3 2QG UK; 4 Institute of Cardiovascular Science, University College London, Gower Street, London, WC1E 6BT UK; 5 Centre for Medical Image Computing, University College London, Gower Street, London, WC1E 6BT UK; 6 Materials Chemistry Research Centre, Department of Chemistry, University College London, London, WC1H 0AJ UK; 7 Photonic Innovations Lab, Department of Electronic and Electrical Engineering, University College London, Roberts Building, London, WC1E 7JE UK; 8 William Harvey Cardiovascular Research Institute, Queen Mary University of London and Barts Health Centre, London, EC1A 7BE UK

## Abstract

Miniaturised high-resolution imaging devices are valuable for guiding minimally invasive procedures such as vascular stent placements. Here, we present all-optical rotational B-mode pulse-echo ultrasound imaging. With this device, ultrasound transmission and reception are performed with light. The all-optical transducer in the probe comprised an optical fibre that delivered pulsed excitation light to an optical head at the distal end with a multi-walled carbon nanotube and polydimethylsiloxane composite coating. This coating was photoacoustically excited to generate a highly directional ultrasound beam perpendicular to the optical fibre axis. A concave Fabry-Pérot cavity at the distal end of an optical fibre, which was interrogated with a tuneable continuous-wave laser, served as an omnidirectional ultrasound receiver. The transmitted ultrasound had a −6 dB bandwidth of 31.3 MHz and a peak-to-peak pressure of 1.87 MPa, as measured at 1.5 mm from the probe. The receiver had a noise equivalent pressure <100 Pa over a 20 MHz bandwidth. With a maximum outer probe diameter of 1.25 mm, the probe provided imaging with an axial resolution better than 50 µm, and a real-time imaging rate of 5 frames per second. To investigate the capabilities of the probe, intraluminal imaging was performed in healthy swine carotid arteries. The results demonstrate that the all-optical probe is viable for clinical rotational ultrasound imaging.

## Introduction

High-frequency ultrasound can provide detailed visualisation of tissue for guidance and diagnostics during minimally invasive surgery^1,2^, for example it has been shown to improve outcomes during coronary artery stent placement^3^. Conventionally, ultrasound devices designed for imaging inside the human body use electronic transducers^4–8^. However, all-optical ultrasound (OpUS) transducers^9–11^ have emerged as promising alternatives. OpUS transducers use pulsed or modulated light to generate ultrasound via the photoacoustic effect^11–16^; ultrasound reflections from tissue are received with optical interferometry^10,17–19^. Miniature OpUS transducers fabricated on optical fibres^9–11,13,15,20^ have several promising features, including broad bandwidths, high sensitivity, and immunity to electromagnetic interference. The broad bandwidths and high sensitivity allow for pulse-echo ultrasound imaging with high axial resolution and large imaging depths, respectively; the immunity to electromagnetic interference allows for compatibility with other medical devices such as radiofrequency ablation catheters^21^. Another promising feature of all-optical techniques is the ability to integrate other optical modalities, such as photoacoustic imaging, via the optical fibres used to transmit and receive ultrasound^22^.

Compared with piezoelectric ultrasound, OpUS is an emerging technology for minimally invasive clinical applications. Whilst OpUS has been previously used for tomographic imaging^23–27^, to date, there have been a small number of studies in which tissue imaging was performed with devices suitable for minimally invasive imaging^9,15,20,22^ and one *in vivo* demonstration^10^. However, in previous demonstrations with fibre-optic OpUS transducers, ultrasound was generated directly ahead of the optical fibres to provide a forward-view along the longitudinal fibre axis. In many clinical contexts, it is desirable to have a side-view from the device that provides the operator with a circumferential image from within a luminal structure. These structures include coronary arteries, branches of the bronchial tree, and bile ducts. Here, for the first time, we present a side-viewing probe with an OpUS transducer that has lateral dimensions suitable for intraluminal rotational scan imaging. Additionally, the presented device was capable of two-dimensional imaging at 5 frames per second, with a real-time display of the data.

Four primary challenges were addressed in this study. First, the OpUS transmitter had to be configured so that the ultrasound beam propagated perpendicular to the optical fibre axis. Second, a low divergence for this beam was required, to allow for imaging with high angular resolution. Third, the OpUS receiver had to receive reflected ultrasound waves that propagated perpendicular to the optical fibre axis. Finally, a method for acquiring B-mode, circumferential images from multiple angles without significantly twisting the optical fibres was needed. Here, we present innovations to overcome these challenges, and demonstrate the translational potential of the OpUS imaging probe with imaging of vascular tissue.

## Results

**Figure 1:**
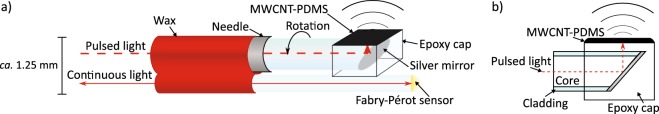
(**a**) Schematic of the side-viewing optical ultrasound transducer showing both the rotating ultrasound transmitting fibre and the stationary ultrasound receiving fibre. (**b**) Cutaway schematic of the fabricated side-viewing optical ultrasound transmitter.

A highly directional optical ultrasound transmitter was paired with an omnidirectional receiver (Fig. 1(a)). This arrangement enabled imaging by rotating only the transmitting fibre; the receiver was stationary. To transmit ultrasound laterally, photoacoustic excitation light was redirected by polishing the optical fibre end surface to 45° and coating it with a mirror (Fig. 1(b)). To achieve a low divergence ultrasound beam, the end of the fibre was encapsulated with optical-grade epoxy in a mould to create a flat ultrasound generating surface with a large aperture (*ca*. 0.5 mm diameter). The flat epoxy surface was coated with an ultrasound-generating composite material comprising functionalised multi-walled carbon nanotubes (MWCNT) and medical-grade polydimethylsiloxane (PDMS)^20^. The ultrasound transmitter was rotated using a custom fibre optic rotary system (see Materials and Methods Section & Supplementary Fig. 4). The omnidirectional ultrasound receiver was a Fabry-Pérot fibre optic sensor with a plano-concave cavity at the distal end. As reported recently, these sensors have been shown to provide high bandwidths and sensitivities (noise equivalent pressure of less than 100 Pa over a bandwidth of 20 MHz), which are essential for high resolution, high depth imaging^10,17^. The OpUS transducer was interrogated and controlled using a custom-built console (see Materials Methods section). Cross-talk signals resulting from ultrasound transmitted directly from the transmitter to the receiver were present at all rotational angles. The cross-talk signal amplitude was dependent on the direction of the transmitter relative to the receiver; it was highest when the transmitter was facing the receiver. High-pass filtering and time-gain compensation were used to reduce the effects of cross-talk signals on imaging.

### Optical ultrasound performance characterisation

**Figure 2:**
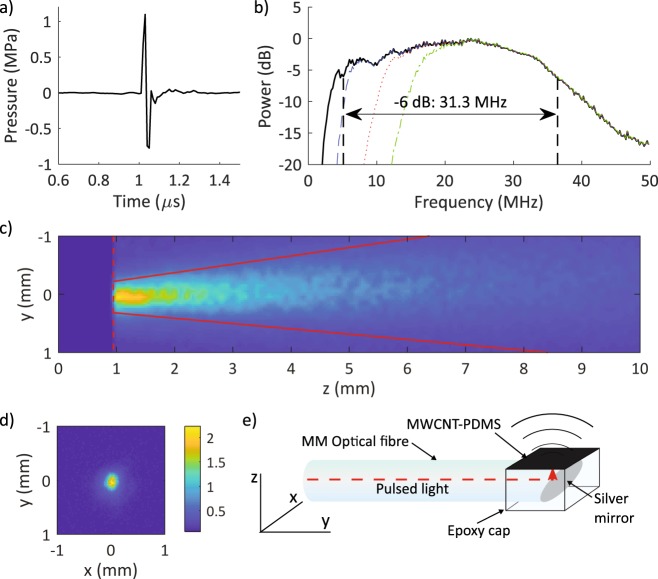
(**a**) Transmitted ultrasound time-series, measured at 1.5 mm and (**b**) the corresponding ultrasound power spectrum, with frequency-filtered power spectra used for the depth-dependent filtering shown (blue dashed line: 5 MHz cut-off; red dotted line: 10 MHz cut-off; green dot-dash line: 15 MHz cut-off). (**c,d**) Transmitted ultrasound beam profiles measured with a hydrophone positioned (**c**) within a plane parallel to the optical fibre axis (red solid line: FWHM) and (**d**) within a plane perpendicular to the optical fibre axis (colour bar in MPa) with measurements performed at 1 mm from the optical fibre tip. (**e**) Schematic of the optical fibre ultrasound transmitter tip with the coordinate system corresponding to beam profiles shown in (**c**,**d**).

The optical ultrasound transmitter was characterised prior to its integration into the OpUS imaging probe. The generated ultrasound pressure was 1.87 MPa at 1.5 mm from the transmitter (Fig. 2(a)), which is consistent with previous high-bandwidth MWCNT-PDMS OpUS devices, including those used for *in vivo* M-mode imaging^10,12,20^. The pulse was bipolar in shape (Fig. 2(a)), unlike those generated by piezoelectric devices that exhibit more ringing due to the bandwidth limitations^28–30^. The corresponding −6 dB ultrasound bandwidth was 31.3 MHz, with a centre frequency of *ca*. 20 MHz (Fig. 2(b)). The generated beam profile was highly collimated, with a full-width half-maximum angular divergence of 13°. At 1 mm, the ultrasound beam profile was elliptical (220 × 170 µm) and slightly wider in the axis of the optical fibre (Fig. 2(c,d)).

With OpUS imaging, the spatial resolution is dependent on several factors, including the transmit and receive bandwidths, and the transmit beam profile. To achieve high axial resolution, high frequencies and bandwidths are necessary. However, imaging with high frequency ranges results in low imaging depths due to the attenuation of ultrasound in tissue. To achieve both high axial resolution imaging at low depths and high penetration in tissue, imaging with both high and low frequency ranges is required^31^. To achieve high angular resolution without synthetic aperture imaging, a focused or collimated ultrasound beam is necessary. The extent to which the beam is focused or collimated tends to increase with frequency and the lateral dimensions of the ultrasound generating region.

**Figure 3:**
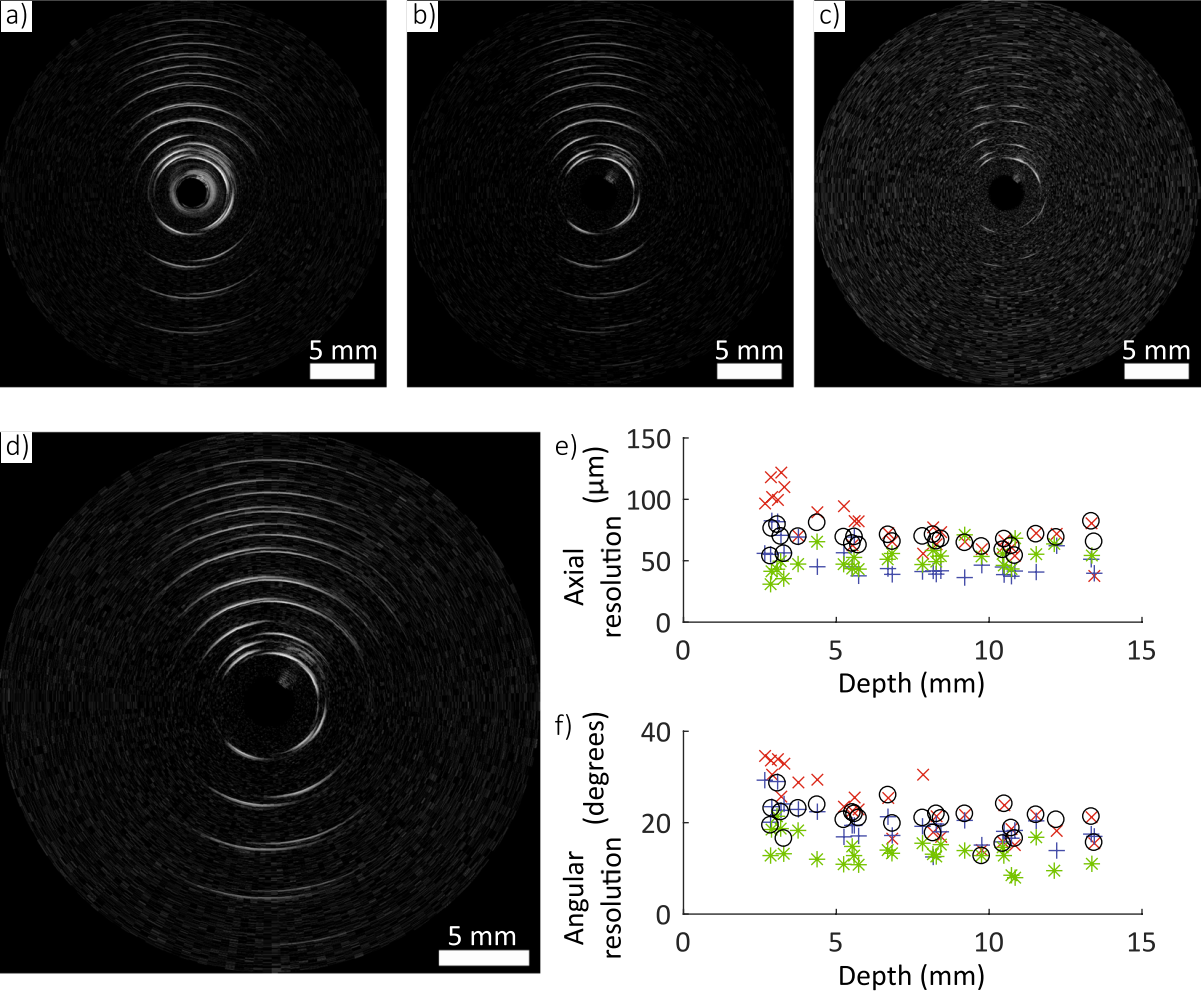
(**a–d**) Rotational optical ultrasound images of a custom 27 µm tungsten wire phantom with different frequency filters: (**a**) 0.5 MHz cut-off, (**b**) 10 MHz cut-off, (**c**) 20 MHz cut-off, (**d**) depth-dependent filter (30 dB dynamic range). (**e**) Axial and (**f**) angular ultrasound resolution for the rotational optical ultrasound (OpUS) imaging probe (Red ×: 0.5 MHz cut-off; blue + : 10 MHz cut-off; green *: 20 MHz cut-off; black o: depth-dependent filter), as measured from the images shown in (**a**–**d**).

Imaging resolution was measured with a custom phantom comprising a series of parallel tungsten wires (27 µm diameter) mounted on a frame (Supplementary Fig. 1). Phantom imaging involved data acquisition followed by processing and interpolation to cartesian co-ordinates (see Materials and Methods section) to generate ultrasound images (Fig. 3). During data processing, different high-pass frequency filter cut-offs were used to assess the impact of the ultrasonic frequency content on the image resolution (Fig. 3(a–c)). For each of the reconstructed images, the axial and angular resolution were calculated as the full-width half-maximum (FWHM) values of the point spread functions generated by the tungsten wires in the images. It was found that increasing the cut-off frequency for the high-pass filter improved the spatial resolution (Fig. 3(e,f)) (Supplementary Table 1). In particular, the axial resolution improved from 122 μm to 71 μm as the cut-off was increased from 0.5 MHz to 20 MHz, and the average angular resolution improved from 23.5° to 13.7°. However, these increases in axial resolution came at the expense of decreases in signal-to-noise that were particularly prominent at larger depths. For a cut-off frequency of 0.5 MHz, both the axial resolution and the angular resolution increased with depth (Fig. 3(e,f)). These increases likely resulted from out-of-plane contributions which are more severe at lower frequencies, where the ultrasound is less confined. To make use of the increase in axial resolution with an increase in cut-off frequency, whilst still maintaining a large imaging depth, a depth-dependent frequency filter was implemented (see Materials and Methods section). With this filter, an axial resolution of 54 μm and an angular resolution of 19.4° were attained at shallow depths (<3 mm). At a larger depth of 10.5 mm, the axial resolution was 59 μm and the angular resolution was 15.4°. These values are comparable to typical commercial intravascular ultrasound (IVUS) devices, which can achieve axial resolutions between 100 and 150 µm depending on the bandwidth and frequency of the device^32,33^. The use of depth-dependent frequency filtering, which is practical with the high bandwidths of the optical transmitter and receiver, could allow for resolving fine features at the luminal surface with high accuracy and imaging objects deep within tissue, beyond the lumen wall.

### Tissue imaging

**Figure 4:**
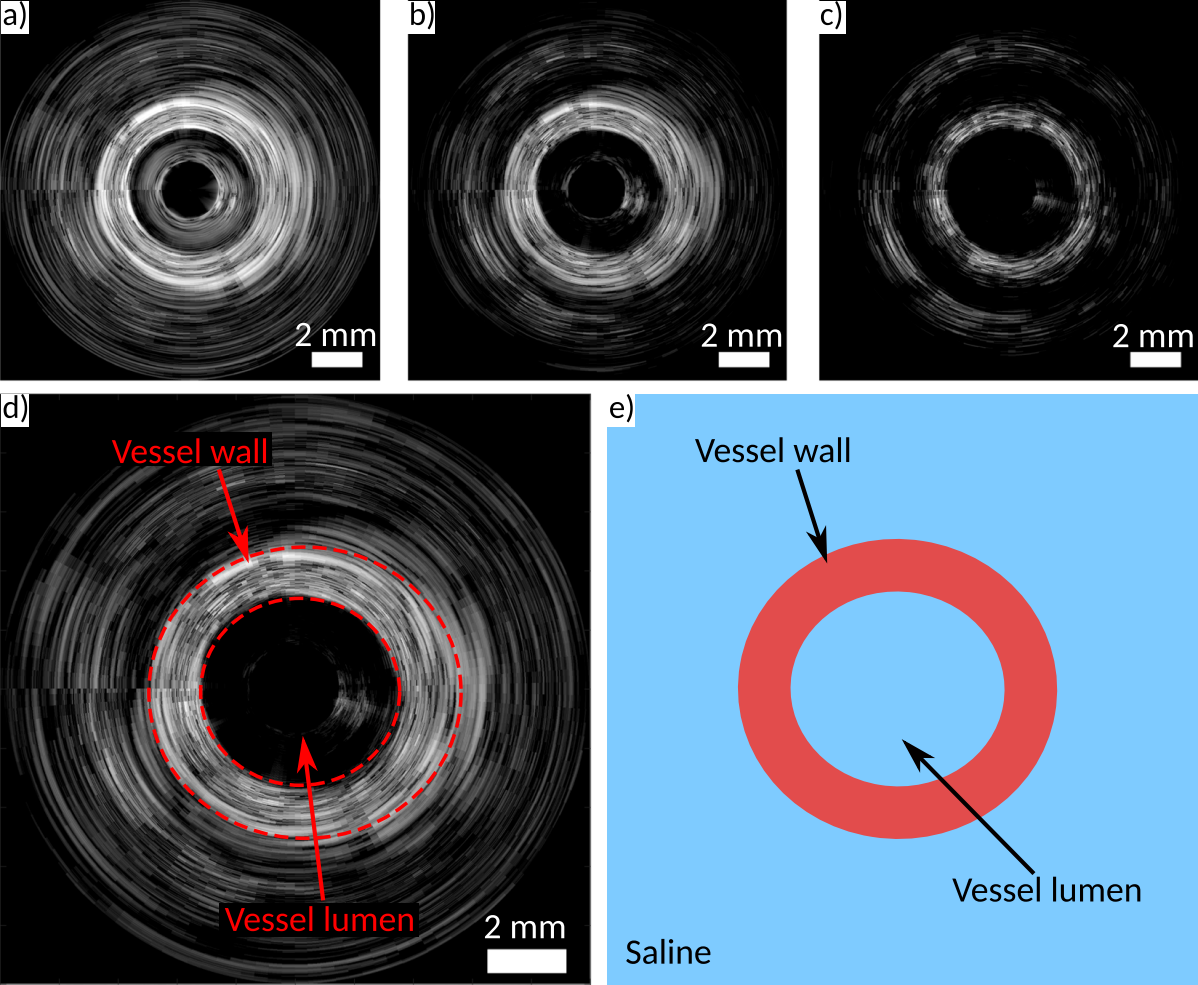
(**a–d**) Rotational optical ultrasound images of an *ex vivo* swine carotid artery with different frequency filters: (**a**) 0.5 MHz cut-off, (**b**) 10 MHz cut-off, (**c**) 20 MHz cut-off, (**d**) depth-dependent filter (40 dB dynamic range). (**e**) corresponding schematic of the imaged vessel section. The full video corresponding to (**a**–**d**) is provided in Supplementary Movie 1.

Vascular tissue *ex vivo* was imaged to investigate the clinical potential of the OpUS probe and demonstrate its capability for intraluminal imaging. Swine carotid arteries were mounted on a custom plastic mount (Supplementary Fig. 3) and rotational imaging was performed using the OpUS probe. The images shown in Fig. 4 corresponded to a still taken from Supplementary Movie 1. The same frequency filtering protocol was applied as with the resolution images. For all frequency filters used, the inner lumen surface was well delineated. However, the shape and detail improved as the high-pass cut-off of the frequency filter was increased (Fig. 4(a–c)). For a high-pass cut-off of 0.5 MHz significant blurring was observed in the rotational aspect (Fig. 4(a)). However, when a high-pass filter set at 20 MHz was used, the full thickness of the vessel could not be resolved (Fig. 4(c)), demonstrating the trade-off between resolution and signal-to-noise. By using a lower frequency cut-off or a depth-dependent filter the full depth of the vessel wall was resolved (Fig. 4(b,d)), whilst maintaining a high depth of penetration. The measured lumen diameter was *ca*. 4 mm (Fig. 4(d)). The acquired data demonstrated the potential for tissue imaging with the OpUS probe.

## Discussion

In this study, we provide the first proof-of-concept that all-optical ultrasound is suitable for B-mode rotational imaging. The OpUS probe demonstrated here is distinct from previous forward-viewing designs, where ultrasound was generated ahead of the optical fibre. Further to this, the system was capable of displaying B-mode images in real-time, with a frame rate of 5 frames per second. The broadband ultrasound signal generated by the probe allowed for both high- and low-frequency imaging of vascular tissue with a dynamic range of 40 dB. Its design could readily be adapted to provide imaging within blood vessels and other luminal bodies. Two key innovations were a rotational side-transmitting, highly-directional optical ultrasound transmitter and its use in combination with an omni-directional optical ultrasound receiver. This rotational OpUS probe contrasts with typical minimally invasive rotational piezoelectric probes, where both transmission and reception are directional.

The spatial resolution of the OpUS imaging probe was sufficient to clearly delineate the vessel lumen and thereby to enable accurate estimation of the vessel inner diameter along different axes. With commercial IVUS probes, axial resolutions ranging from 100 to 150 µm and lateral resolutions ranging from 150 to 300 µm have been reported^32,33^. Recently, dual-frequency probes with axial resolutions ranging from 16 to 80 µm have been reported^34^. In future probes, the lateral resolution could be improved with the use of a concave ultrasound generating surface to achieve a tighter focus, as previously demonstrated with forward viewing OpUS^35^ or by increasing the aperture of the ultrasound transmitter. In addition to improving the lateral resolution, reducing the ultrasound beam divergence can be expected to improve the signal-to-noise ratio for a given depth, and thereby to increase the imaging depth in tissue. The axial resolution could be improved by increasing both the transmitted and received ultrasound frequency. The transmitted frequency could be optimised by adjusting the excitation light pulse duration, or by using light pulses or chirps of different durations^13^. Likewise, the received bandwidth could be increased by decreasing the thickness of the Fabry-Pérot cavity, albeit at the expense of decreased sensitivity^9^. As post-processing steps, more complex filtering and reconstruction strategies, such as adapting the frequency cut-offs to image features, could be used in combination with speckle-reduction filters^36^ and acoustic clutter reduction algorithms^37^. For example, for high-resolution, low penetration depth applications, such as assessing stent apposition in cardiovascular interventions, a high frequency cut-off could be used to improve the resolution. Likewise, with the same probe, a low frequency cut-off would provide a large penetration depth to assess plaque thickness. More generally, adaptive frequency filtering could be used to accommodate acoustically heterogeneous tissues^38,39^.

With the paradigm for rotational OpUS imaging presented here, significant increases in the image acquisition rate are possible. Currently, this rate is limited to 5 frames per second by the data transfer rate and computation times, compared to 30 frames per second for commercially available intravascular ultrasound imaging catheters^32^. However, the transmitter can provide pulses at a repetition rate of 8 kHz, with the same excitation light pulse energies used in this study, provided that there is sufficient cooling (data not shown). Thus, if the acquisition rate was solely limited by the transmitter repetition rate, the device could provide images at rates >100 frames per second. To perform imaging of vessels deep within the body *in vivo*, lengthening the OpUS imaging probe will be required. A torque coil similar to those used in commercial IVUS and optical coherence tomography (OCT) catheters could enable high imaging rates without undue twisting of the transmit optical fibre.

The images presented here demonstrate the feasibility of OpUS imaging to provide visualisation within luminal bodies. The high bandwidths and sensitivities that can be achieved allow for high spatial resolution and high imaging depths, which would be beneficial for many clinical applications that extend beyond the vascular system, including cervical and bronchial imaging. The absence of electronic components lends itself to MRI compatibility, which would be beneficial for guiding minimally invasive neuro interventions such as deep brain stimulation. Additionally, immunity to electromagnetic interference would be beneficial to visualise radiofrequency ablations in real-time. Within intravascular imaging there has been significant recent interest in using hybrid imaging techniques to supplement microstructural information with molecular contrast^1,40–42^, such as that provided by photoacoustic imaging^43–48^ or fluorescence sensing^49–51^. The probe presented in this study could be readily extended to provide dual-modality photoacoustic and ultrasound imaging using wavelength-selective coatings such as those developed from PDMS and gold nanoparticles^22^. We conclude that rotational scan OpUS imaging is a viable and promising paradigm for guiding minimally invasive procedures and this study represents the first steps towards future clinical devices.

## Materials and Methods

### Optical ultrasound receiver

The optical fibre used for ultrasound reception was single-mode (SMF-28) with core/cladding diameters of 8/125 μm. A Fabry–Pérot (FP) cavity was created at the distal end by dip coating into an optically transparent polymer, as described previously^17^. Two dielectric mirrors were deposited before and after dip coating: one on the optical fibre end face and a second on the outer surface of the polymer. Both mirror reflectivities were nominally 98% in the range of 1500–1600 nm. The FP cavity was coated in a protective layer of parylene C of approximately 5 μm in thickness.

### Optical ultrasound transmitter

The multi-mode optical fibre used for ultrasound generation had silica core/cladding diameters of 400/440 μm (CeramOptec GmbH, Germany) and a polyimide buffer coating with a diameter of 470 µm. The buffer layer was stripped from the last 10 mm of the optical fibre using warm sulphuric acid. Subsequently, the bare glass end was polished to 45° using a fibre polishing system (KrellTech, NJ, USA). A silver mirror was applied to the polished surface by manually brushing with silver paint (186–3600, RS Pro, UK). The silver paint was left to cure for *ca*. 12 hours. Subsequently, a UV curable optical epoxy cap (NOA81, Norland Products, UK) was applied to the distal end (width × length × depth *ca*. 0.6 × 7.5 × 0.6 mm) using a custom polydimethylsiloxane (Sylgard 184) mould. An MWCNT coating described previously^20^ was applied to the optically emitting surface of the epoxy. Finally, the entire distal end was dip-coated in a solution of PDMS and xylene (1:1) and left to cure for *ca*. 24 hours.

### Console

The console comprised optoelectronic components to deliver pulsed excitation light to the OpUS transmitter and to interrogate the OpUS receiver. For ultrasound transmission, pulsed light was delivered into the ultrasound generating fibre from a Q-switched Nd:YAG laser (SPOT-10-500-1064, Elforlight, UK). This laser had a wavelength of 1064 nm, a pulse width of 2 ns and a pulse energy of 40 µJ. For ultrasound reception, continuous-wave light was delivered into the ultrasound receiving fibre from a tuneable laser (Tunics T100S-HP CL, Yenista Optics, France), via a circulator. This laser had a wavelength range of 1500–1600 nm and an output power level of 4.5 mW. The reflected optical signal was received using a custom photoreceiver which split the signal into low (<50 kHz) and high (>500 kHz) frequency components. The low frequency component was digitised at 16 bits with a sample rate of 1 MS/s and used to acquire the interferometer transfer function of the sensor and identify the optimum bias wavelength. The high frequency component was digitised at 14 bits with a sample rate of 100 MS/s and was encoded with the received ultrasound signals.

### Data acquisition

The FP sensor was affixed to the outside of a 21-gauge hypotube using sealing wax. The ultrasound transmitting fibre was inserted through the hypotube, aligned longitudinally with the fibre-optic ultrasound sensor such the that transmitting element was <1 mm proximal to the receiving element. The transmitter was held in custom built fibre optic rotary system (Supplementary Fig. 4), which consisted of a custom fibre optic rotary junction (Princetel, US), built into a housing for stability. Using this method, only the transmitter was rotated whilst the Fabry-Pérot sensor was held stationary on the outside of the metal hypotube. Rotation was driven using a stepper motor (ST2818M1006-B, Nanotec, Germany). This system was capable of imaging at 5 frames per second. Rotational imaging was performed by continuously rotating the transmitting fibre and acquiring data at 5° increments. For each A-line acquired, 1000 sensor data points were recorded, which corresponded to a depth of *ca*. 7.5 mm. Images were converted from polar to Cartesian co-ordinates and displayed in real time during imaging to enable positioning of the device within the image target.

### Image processing

Depth-dependent filtering was applied to the acquired image data. Here, the data was high pass filtered (4^th^ order Butterworth) with a cut-off frequency that varied with depth (Supplementary Fig. 2). Briefly, the filter cut-off linearly decreased from a value of 15 MHz at a depth of 0 mm to 1 MHz at 10 mm. For depths greater than 10 mm, the cut-off frequency was fixed at 1 MHz. Following depth-dependent filtering, a median filter was applied to reduce the speckle and the signal envelope was found using the absolute value of the Hilbert transform, followed by log transformation. Subsequently, time-gain compensation was applied, as previously described^10^, to suppress direct ultrasound transmission from the transmitter to the receiver. Finally, the data was interpolated from polar to cartesian co-ordinates for display.

### Vascular tissue imaging

Vascular imaging targets comprised swine carotid arteries that were mounted in plastic housings (Supplementary Fig. 3). The swine vessels (Medical Meat Supplies, UK) had been frozen prior to use. The plastic housings had holes to provide access to the vessel lumens. The vessels were glued such that the vessel lumen was aligned with the holes on the housing.

## Supplementary information

Supplementary Movie 1

Supplementary Information

## Data Availability

The datasets generated during and/or analysed during the current study are available from the corresponding author on reasonable request.
