# Supplementary material for: All-Optical Rotational Ultrasound Imaging
Source: Sci Rep. 2019 Apr 3;9:5576. doi: 10.1038/s41598-019-41970-z (PMC6447544; doi:10.1038/s41598-019-41970-z)
Supplement: Supplementary file 2 — Supplementary Information [file 41598_2019_41970_MOESM2_ESM.pdf]

Supplementary Information

All-Optical Rotational Scan Ultrasound Imaging

Richard J. Colchester[1,2], Callum Little[2,3,4], George Dwyer[1,2,5], Sacha Noimark[1,2,6], Erwin J. Alles[1,2], Edward Z. Zhang[1], Chris D. Loder[3,4], Ivan P. Parkin[6], Ioannis Papakonstantinou[7], Paul C. Beard[1,2], Malcolm C. Finlay[8], Roby D. Rakhit[3,4], Adrien E. Desjardins[1,2]

[1]Department of Medical Physics and Biomedical Engineering, University College London, Malet Place Engineering Building, London, WC1E 6BT, UK

[2]Wellcome/EPSRC Centre for Interventional and Surgical Sciences, University College London, Charles Bell House, 67-73 Riding House Street, London, W1W 7EJ, UK

[3]Department of Cardiology, Royal Free Hampstead NHS Trust, Pond Street, London NW3 2QG, UK

[4]Institute of Cardiovascular Science, University College London, Gower Street, London, WC1E 6BT, UK

[5]Centre for Medical Image Computing, University College London, Gower Street, London, WC1E 6BT, UK

[6]Materials Chemistry Research Centre, Department of Chemistry, University College London, London, WC1H 0AJ, UK

[7]Department of Electronic and Electrical Engineering, University College London, Roberts Building, London, WC1E 7JE, UK

[8]William Harvey Cardiovascular Research Institute, Queen Mary University of London and Barts Health Centre, London, EC1A 7BE, UK

1. Resolution Phantom

A resolution phantom was made by winding a continuous piece of tungsten wire over an acrylic frame to produce a series of parallel wires.

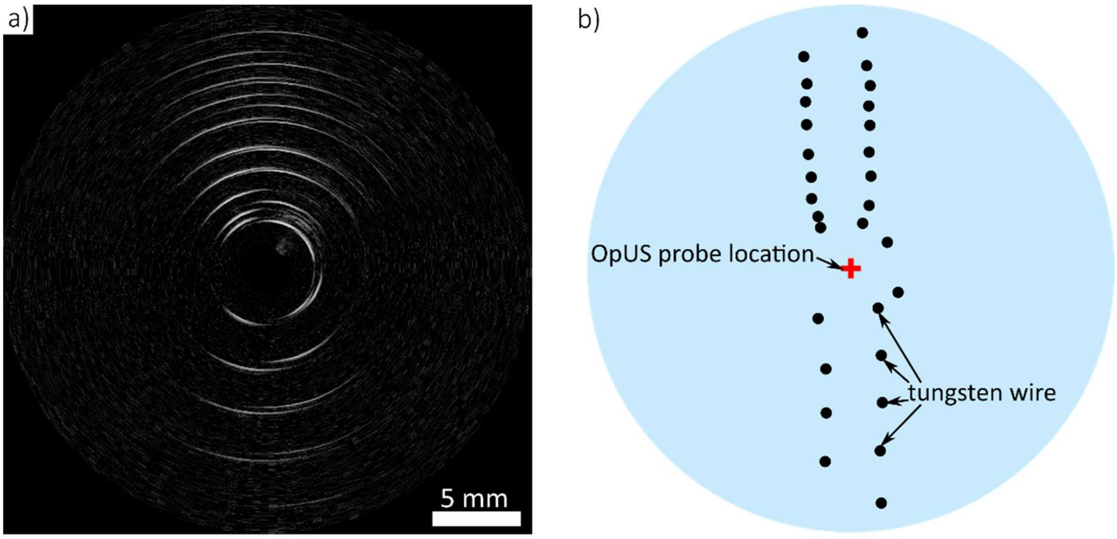

Figure 1. (a) Rotational optical ultrasound image of a custom 27 µm tungsten wire phantom with using a depth-dependent filter (30 dB dynamic range). (b) Schematic of the tungsten wire phantom corresponding to (a), showing the location of the OpUS probe and the tungsten wires.

2. Depth Dependent Frequency Filtering

Prior to further processing a depth-dependent frequency filter was applied to the acquired data. A high-pass frequency filter was applied (4th order Butterworth) where the frequency cut-off was decreased linearly with increasing depth, from a value of 15 MHz at 0 mm to 1 MHz at 10 mm (Figure 1). For depths greater than 10 mm, the cut-off was kept at a constant value of 1 MHz. These values for the filter were chosen empirically, by visual inspection of the generated images.

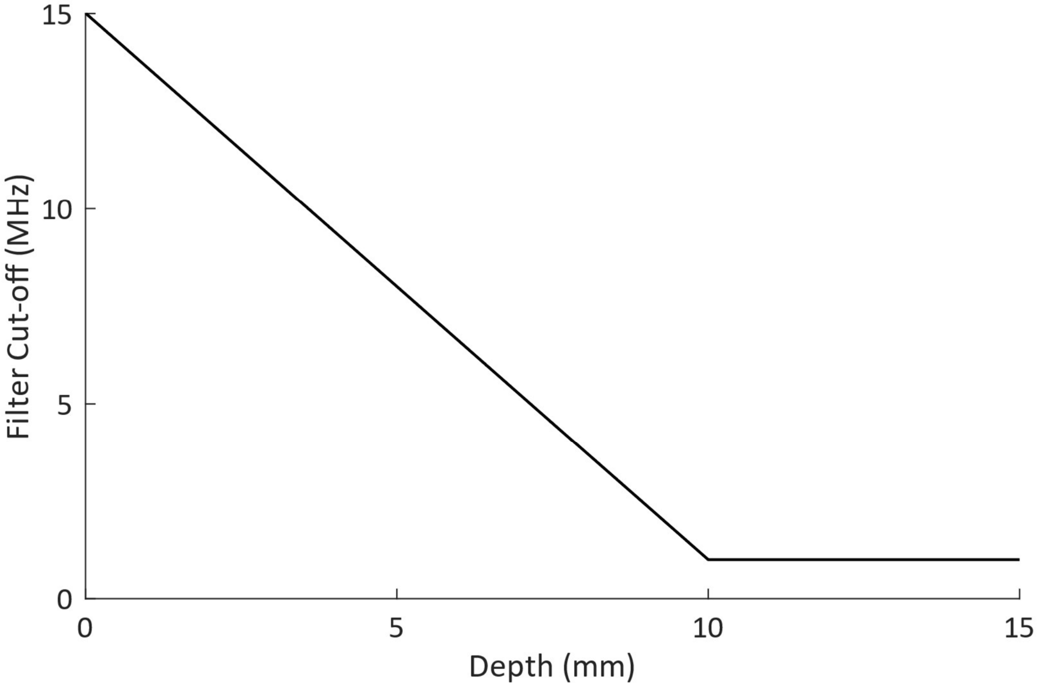

Figure 2. Depth-dependent filter cut-off used during image processing.

3. Imaging Resolution

Filter	Axial Resolution (µm)			Angular Resolution (°)		
	**Maximum**	**Minimum**	**Average**	**Maximum**	**Minimum**	**Average**
High Pass; 0.5 MHz cut-off	**122**	**38**	**77**	**34.6**	**14.0**	**23.5**
High Pass; 10 MHz cut-off	**83**	**36**	**50**	**29.0**	**12.5**	**19.6**
High Pass; 20 MHz cut-off	**71**	**31**	**51**	**21.4**	**8.0**	**13.7**
Depth-Dependent Filter	**88**	**37**	**55**	**26.4**	**13**	**19.1**

Table 1. Maximum, minimum and mean resolution values as measured using different temporal filters on the received data.

4. **Vessel Mounting**

A custom vessel mount was made using a plastic construction kit (Lego). A box was made to contain the gelatine backing, with a hole at each end. The vessel was mounted across the holes such that its lumen could be accessed from outside the box. The vessel was affixed to the mount using adhesive (Universal Super Glue, Loctite, UK).

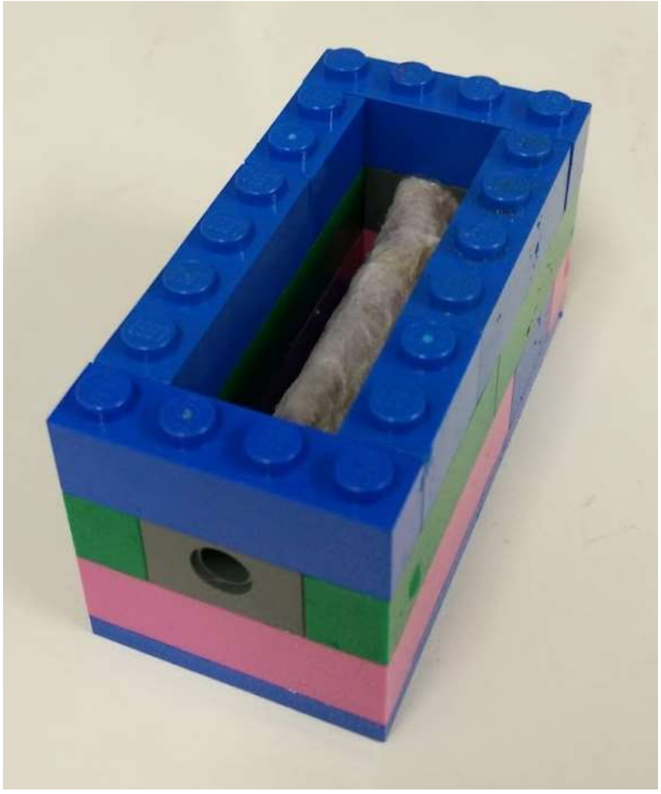

Figure 3. Photograph of a swine carotid affixed in a custom mount.

5. Custom Fibre Optic Rotary System

a)

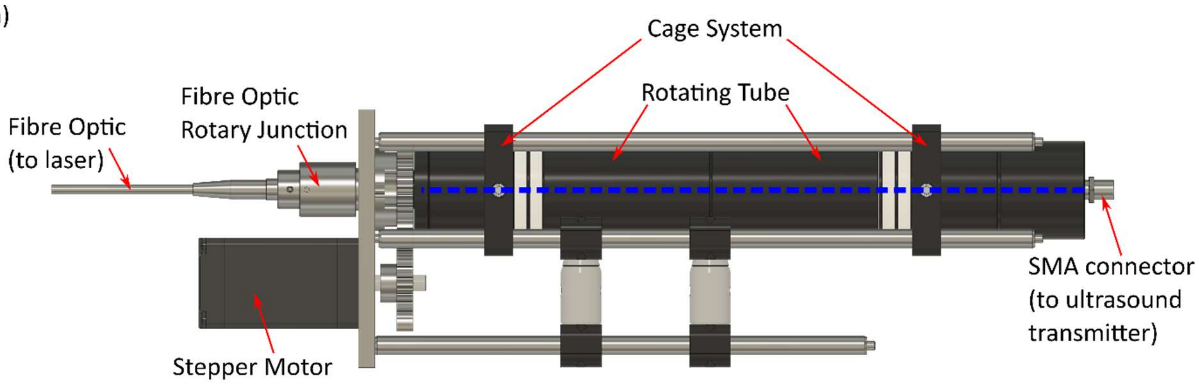

b)

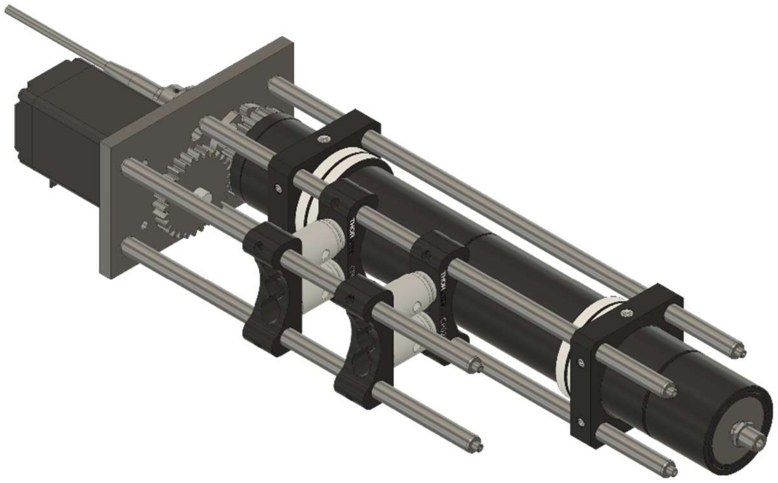

Figure 4. (a) Top down view of the CAD design for the custom fibre optic rotary system used to rotate the side-viewing fibre optic ultrasound transmitter. The rotation was driven by a stepper motor (Nanotec, Germany) and a fibre optic rotary junction (Princetel, US) was used to allow the transmitter fibre to rotate independently of the fibre optic patch cable connected to the pulsed excitation laser. A cage system was used to hold a tube which contained a fibre optic patch cable (blue dashed line). This constrained the fibre optic cable in the axial and lateral dimensions during rotation. The transmitter fibre was connected to the SMA connector at the end of the rotating tube. (b) Angled view of the CAD design for the custom fibre optic rotary system.
